# Benchmarking integrated linear-optical architectures for quantum information processing

**DOI:** 10.1038/s41598-017-15174-2

**Published:** 2017-11-09

**Authors:** Fulvio Flamini, Nicolò Spagnolo, Niko Viggianiello, Andrea Crespi, Roberto Osellame, Fabio Sciarrino

**Affiliations:** 1 Dipartimento di Fisica, Sapienza Università di Roma, Piazzale Aldo Moro 5, I-00185 Roma, Italy; 2 Istituto di Fotonica e Nanotecnologie, Consiglio Nazionale delle Ricerche (IFN-CNR), Piazza Leonardo da Vinci, 32, I-20133 Milano, Italy; 3 Dipartimento di Fisica, Politecnico di Milano, Piazza Leonardo da Vinci, 32, I-20133 Milano, Italy

## Abstract

Photonic platforms represent a promising technology for the realization of several quantum communication protocols and for experiments of quantum simulation. Moreover, large-scale integrated interferometers have recently gained a relevant role in quantum computing, specifically with Boson Sampling devices and the race for quantum supremacy. Indeed, various linear optical schemes have been proposed for the implementation of unitary transformations, each one suitable for a specific task. Notwithstanding, so far a comprehensive analysis of the state of the art under broader and realistic conditions is still lacking. In the present work we fill this gap, providing in a unified framework a quantitative comparison of the three main photonic architectures, namely the ones with triangular and square designs and the so-called fast transformations. All layouts have been analyzed in presence of losses and imperfect control over the internal reflectivities and phases, showing that the square design outperforms the triangular scheme in most operational conditions. Our results represent a further step ahead towards the implementation of quantum information protocols on large-scale integrated photonic devices.

## Introduction

Several milestone achievements in experimental quantum information are pushing the limits of integrated photonic technologies in numerous relevant applications. Single-photon sources^1–4^ and detectors^5,6^ are already providing remarkable results in first benchmark demonstrations, while a number of powerful techniques have been developed to fully characterize general quantum processes^7–12^. The miniaturization of complex interferometric schemes is thus expected to unlock stable and mass-produced large-scale quantum information protocols, among the others for teleportation^13^, logic gates^14,15^, quantum networks^16,17^ and light manipulation^18,19^. One further, fundamental feature of such platforms is the capability of adding dynamical reconfigurability to the circuits^20–23^, allowing for universal applications as for standard classical processors^24–26^. More in particular, a research area that well benefits from all the above-mentioned technologies is that of Boson Sampling^27–35^, where efficient sampling from linear photonic devices is expected to provide evidence of a quantum computational power beyond the reach of classical computers^35–37^.

In this context, it is essential to identify suitable architectures to implement large-size interferometric networks within an integrated platform. In ref.^38^, Reck *et al*. proposed a universal algorithm to implement an arbitrary unitary transformation by decomposing it in a suitable network of unit cells made up of only beam splitters and phase shifters. For each transformation to be implemented, it is sufficient to determine the correct set of parameters (beam splitter transmittivities and internal phases) without altering the overall interferometric layout. This architecture has been employed in first experimental instances of Boson Sampling^30^, where the capability to implement arbitrary Haar random unitaries is an essential ingredient for the demonstration of its computational complexity. However, this architecture lacks a perfect symmetry in its triangular layout, thus making it sensitive to internal losses that lower the adherence of the implemented transformation to the ideal one. Recently, two different architectures have been proposed for the implementation of linear optical networks. A first scheme has been reported in ref.^39^, which ultimately corresponds to a cunning rearrangement of the scheme of ref.^38^ in a symmetric layout. While keeping the same number of optical elements and the capability of implementing an arbitrary unitary transformation, this scheme presents reduced sensitivity to losses within the interferometer. A second scheme, inspired by the classical algorithm of Cooley and Tukey^40^ for the fast Fourier transform, has been proposed in refs^41,42^ and implemented experimentally in refs^43–46^ by exploiting the three-dimensional capabilities of femtosecond laser micromachining^47,48^. This layout, though not supporting arbitrary unitary evolutions, allows to implement a significant class of linear optical networks with a substantial reduction in the number of necessary optical elements. Such class of matrices includes the Hadamard ones, with a notable example provided by the Fourier transformation that is widely employed in a large set of quantum information protocols^49,50^. A crucial requirement towards the identification of optimal architectures for the implementation of large size interferometers is a detailed knowledge of the tolerance to fabricative errors, namely propagation losses and imperfections in the parameters of the optical elements. Indeed, in all quantum information applications including Boson Sampling^51–56^ the applicability of a given experimental platform is limited by the maximum amount of noise tolerable in the interfometric networks. Within this general framework, a thorough analysis of the tolerance of the proposed architectures in the presence of fabrication noise is still lacking.

**Figure 1:**
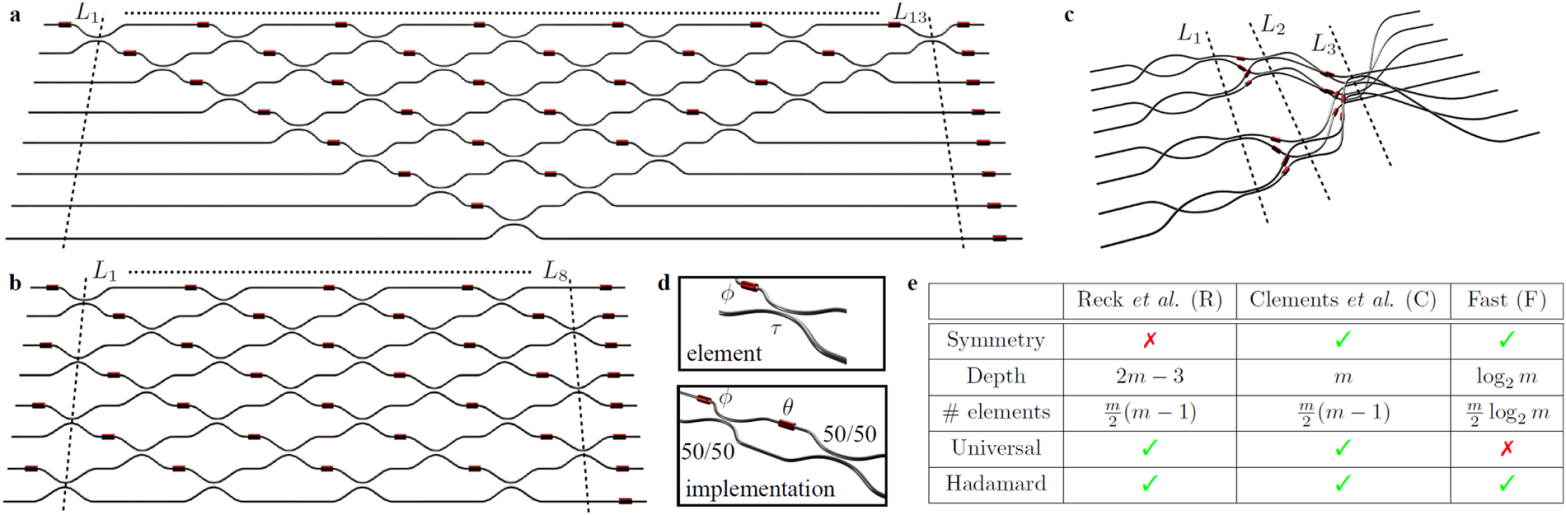
Architectures for integrated photonic networks. Interferometric layouts for a 8-mode unitary transformation with (**a**) the triangular scheme of Reck *et al*.^38^ and with (**b**) the scheme of Clements *et al*.^39^. For both architectures, capable to implement arbitrary Haar-random transformations, unit cells consist of one beam splitter with arbitrary transmittivity *τ* and one phase shift *ϕ* (red cylinders). (**c**) Fast architecture with 3-dimensional layout shown for a 8-mode interferometer, which realizes a significant class of transformations with a reduced number of layers and optical elements. (**d**) Unit cell for the three layouts. Beam splitters in each unit cell can be implemented as a Mach-Zehnder interferometer composed by two symmetric (50/50) beam splitters and an internal relative phase shift *θ*. (**e**) Table summarizing the main features of the three architectures.

In this article we bridge this gap, presenting a complete analysis of the performance of the main photonic architectures under imperfect operational conditions. The three interferometric layouts have been investigated in the general case, by admitting different levels of losses and noise in unitary transformations of increasing size. Specifically, following the approach commonly adopted for reconfigurable quantum circuits^24,25^, i.e. by modelling beam splitters as Mach-Zehnder interferometers with variable phases and two cascaded symmetric beam splitters, noise was added to their reflectivities and to phases in both Mach-Zehnders and outer phase shifters. For our numerical benchmark we employ as figures of merit the fidelity^39,52,55,56^ and the total variation distance (TVD)^53–56^, as good estimators of the distance between ideal and imperfect implementations in relevant applications. In particular, while depending also on external factors like purity and degree of distinguishability between the input photons, the TVD has been already identified as a key figure of merit for the goodness of multiphoton experiments^53–56^ (see Supplementary Note 3). The article is structured as follows. First, we briefly discuss the interferometric structure of the triangular, square and fast designs, whose main characteristics are outlined in Fig. 1. Thereafter, we compare the performances of the first two schemes, which were shown to be universal for unitary evolutions, for the implementation of Haar-random transformations of increasing size. Finally, we compare the operation of the three schemes for the implementation of Fourier and Sylvester interferometers, which represent the fundamental building blocks in a significant number of relevant quantum information protocols. Our analysis highlights the advantages and limitations of each scheme, providing an essential reference point for the design of future larger-scale photonic technologies, whose optimal configurations may well benefit from a joint integration.

## Results

### Designs for photonic architectures

Since the seminal work of Hurwitz^57^, it is known that every *m* × *m* unitary transformation can be decomposed in the action of $\frac{m(m-1)}{2}$ unitaries, each acting on a two-dimensional subspace of the Hilbert space. Reck *et al*.^38^ independently gave the first operational proof (*R*) that an actual (linear optical) implementation, consisting of only single-mode phase shifters and two-mode beam splitters, does exist for any discrete unitary operator. Recently, a new algorithm (*C*) for the decomposition of arbitrary unitary transformations has been introduced by Clements *et al*.^39^, which basically presents a higher resilience against propagation losses thanks to the compact and fully symmetric design. Both *R* and *C* decompositions are made up of a set of $\frac{m(m-1)}{2}$ unitaries ${T}_{k,k+1}^{(i)}$, each coupling step by step modes *k* and *k* + 1 of the interferometer

1 $$\begin{array}{cc}{T}_{k,k+1}^{(i)}=(\begin{array}{cccc}1 &  &  & 0\\  & -\sin \,{\omega }_{i} & {e}^{-\iota {\varphi }_{i}}\,\cos \,{\omega }_{i} & \\  & \cos \,{\omega }_{i} & {e}^{-\iota {\varphi }_{i}}\,\sin \,{\omega }_{i} & \\ 0 &  &  & 1\end{array}), & \qquad \qquad \qquad {U}_{R,C}={\prod }_{i=1}^{\frac{m(m-1)}{2}}{T}_{k,k+1}^{(i)}\end{array}$$

where the order of the interactions is directly related to the triangular and square designs of, respectively, the *R* and *C* schemes. While being both universal for the decomposition of unitary transformations, the *C*-design presents some immediate advantages in terms of circuit depth, namely a more balanced mixing of the optical modes and less propagation losses thanks to a minimized operation area, which is also a crucial requirement for large-scale implementations.

The universality of the *C*- and *R*-designs for unitary decomposition comes however at the cost of ignoring possible symmetries of the transformations, which could reduce the complexity of specific implementations. A relevant example is represented for instance by the Hadamard transformations, whose symmetries are known to allow remarkable simplifications in their algorithmic construction^40^. Notable representatives of the Hadamard class are the Sylvester (*U* ^*S*^) and Fourier (*U* ^*F*^) transformations, described by 2^*n*^-dimensional unitary matrices given by

2 $$\begin{array}{cc}{U}^{S}{(2}^{n})=S{(2}^{n})=(\begin{array}{cc}S{(2}^{n-1}) & S{(2}^{n-1})\\ S{(2}^{n-1}) & -S{(2}^{n-1})\end{array}), & \qquad \qquad {U}_{a,b}^{F}{(2}^{n})=\frac{1}{\sqrt{{2}^{n}}}\,{e}^{2\pi i{\textstyle \tfrac{ab}{{2}^{n}}}}\end{array}$$

being *S*(2^0^) = (1) and *n* any positive integer. More generally, starting from the linear-optical fast Fourier decomposition developed by Barak *et al*.^41,42^, it is possible to generalize their scheme to span a whole class of generalized Hadamard transformations^58^ by keeping fixed the interferometric structure and by tuning the parameters of the beam splitters and phase shifters. The essential structure of such fast architectures presents some interesting advantages with respect to the other universal schemes. First, the depth of the circuit scales only logarithmically with the size of the interferometer, i.e. with the number of optical modes, leading to an even more compact operation area and to reduced propagation losses. Moreover, the layout is fully symmetric and naturally fits a description in terms of qubit states, thanks to the binary interactions between the modes. A closed-form expression of the element *U*_*a*,*b*_ of the most general 2^*n*^-dimensional fast unitary transformation has the form

3 $${U}_{\,a+\mathrm{1,}\,b+1}^{(n)}={e}^{i{b}_{r}{\varphi }_{r}^{{\xi }_{r}^{(a,b)}}+i\tfrac{\pi }{2}{a}_{r}^{(n)}\oplus {b}_{r}^{(n)}}\,\prod _{s=1}^{n}\cos \,{\chi }_{a,b}^{(n,s)}$$

where *a*, *b* ∈ [0, 2^*n*^ − 1] label the input/output modes and some shorthand notations have been used, following the Einstein summation convention

4 $$\begin{array}{cc}{\chi }_{a,b}^{(n,s)}={\theta }_{s,{f}_{s}^{(a,b)}}\,-\,\frac{\pi }{2}\,|{a}_{s}^{(n)}-{b}_{s}^{(n)}|, & {\xi }_{r}^{(a,b)}=1+{2}^{n-r}+b\,{\rm{m}}{\rm{o}}{\rm{d}}\,{2}^{n-r}+\lfloor {\textstyle \tfrac{a}{{2}^{n-r+1}}}\rfloor \,{2}^{n-r+1}\end{array}$$

where ${f}_{s}^{(a,b)}=1+b+({a}_{r}^{(n)}\,-\,{b}_{r}^{(n)})\,{2}^{n-r}$ and ${m}_{r}^{(n)}$ equals the *r*-th digit of the *n*-bit binary representation of *m*, being $\lfloor x\rfloor$ the integer part of the real number *x*.

In general, any *m*-dimensional photonic architectures can be described in terms of consecutive layers *s* of optical elements *L*_*s*_, made up of a network of phase shifters and beam splitters mixing a subset of modes no more than once each. Specifically, each matrix *L*_*s*_ consists in turn of a layer *B* ^(*s*)^ of beam splitters, coupling a set of pairs of modes (*k*_1_, *k*_2_), and $\tfrac{m}{2}$ phase shifters ${e}^{i{\varphi }_{s,{k}_{1}}}$ placed for each pair on one of the two interacting modes (*k*_1_, *k*_2_). The particular sequence of mode interactions {(*k*_1_, *k*_2_)} depends on the triangular, square or fast designs. While clearer for the first two, the geometry of the third scheme for a 2^*n*^-dimensional unitary transformation is slightly more complex and arises from the binary representations of the optical modes. Using *τ*_*s*,*k*_ for the beam splitters transmissivities on mode *k*, the beamsplitters layer is described by the matrix

5 $${B}_{{k}_{1},{k}_{2}}^{(s)}\equiv \{\begin{array}{ll}{\tau }_{s,{k}_{1}} & {k}_{1}={k}_{2}\\ i\,{(1-{\tau }_{s,{k}_{1}}^{2})}^{\mathrm{1/2}} & ({k}_{1},{k}_{2})\in {\{(\alpha ,\beta )\}}^{(n,s)}\\ 0 & otherwise\end{array}$$

where {(*α*, *β*)}^(*n*,*s*)^ = {(*a* + 2^*s*^ *b*, *a* + 2^*s*^ *b* + 2^*s*−1^)} are the pairs of modes interacting in the layer *s*, with *a* ∈ {1, …, 2^*s*−1^}, *b* ∈ {0, …, 2^*n*−*s*^ − 1}. For example, the 8-dimensional quantum Fourier transform is obtained, modulo a relabeling of the output modes^42^, by choosing ${\tau }_{s,k}=\sqrt{{2}^{-1}}$ and ${\varphi }_{\mathrm{2,7}}={\varphi }_{\mathrm{2,8}}={\varphi }_{\mathrm{3,4}}=\tfrac{\pi }{2}$, ${\varphi }_{\mathrm{3,6}}=\tfrac{\pi }{4}$ and ${\varphi }_{\mathrm{3,8}}=\tfrac{3\pi }{4}$. Similarly, the Sylvester transformation corresponds to the choice ${\tau }_{s,k}=\sqrt{{2}^{-1}}$ and *ϕ*_*s*,*k*_ = 0.

### Modelling non-ideal unitary implementations

We can now introduce the model adopted to probe the three architectures under non-ideal conditions. In the following we will refer to the *C*- (Clements *et al*.^39^), *R*– (Reck *et al*.^38^) and *F*- (Fast) designs looking at their fixed, solid photonic architectures. This aspect is especially relevant if we are to choose the layout of a fully reconfigurable quantum circuit, which is designed to be multi-purpose and optimal when averaging over all its applications of interest. In general, the two main factors affecting the implementation of photonic quantum circuits are propagation losses and imperfect settings of the parameters describing the optical elements.

#### Losses

Coupling losses at the input/output of any circuits remains a relevant aspect in practical situations; however, their effect can be regarded as independent of the internal photonic architecture adopted and, thus, they will not be included in our study. Propagation losses, occurring in both straight and bent waveguides, play instead the main role in spoiling multipath interference. Their impact was shown to be mitigated by a more compact and symmetric interferometric structure^39^, where optical modes interact with each other in a balanced way. Though photon losses unavoidably occurr all along the circuit, a simple and effective way to analyze their effect is that of inserting costant losses at the output of each two-mode unit cell.

#### Fabricative noise

Imperfect control over the fabricative parameters naturally leads to deviations from the ideal evolution on the circuit while keeping the unitarity of the process. The source of noise, arising from imperfect fabrication of the beam splitters and phase shifters associated to the ${T}_{k,k+1}^{(i)}$ in Eq. (1), has a different nature depending on the practical realization. Recent technological achievements have enabled the realization of reconfigurable quantum circuits^24,25^, where generic beam splitters are implemented as Mach-Zehnder interferometers with two cascaded symmetric beam splitters and one tunable phase shift. In this case, which will be at the core of our analysis, noise arises from imperfect fabrication of the fixed symmetric beam splitters and from a non-perfect control over the thermo-electric or electro-optic phase manipulation, which becomes non-negligible for large-scale circuits or with high-speed tunings. More specifically, fabrication errors may have a systematic component and a completely random one. Systematic components can be compensated in principle by fabricating several devices, within the same substrate and different geometrical parameters. The use of thermo-optic phase shifters further allows to compensate for all fixed phase imperfections.

To focus our study around realistic values for the above mentioned sources of imperfections, we will consider femtosecond laser writing as the reference fabrication technology, as it is the only one capable to realize all the three considered architectures. In this case, typical bending losses with respect to straight waveguides are of the order of 0.5 dB/cm, which corresponds to 0.2 dB per directional coupler. Realistic values for fabrication errors at each directional coupler are of 0.21 rad for the phase shifters^59^ and about 1–2% for the beam splitter transmissivities. These values do not change significantly considering a 2D or 3D architecture and, concerning phase shifts, a quite higher accuracy down to about 0.01 rad can actually be reached with an active control through thermo-optic phase shifters^23^. Therefore, we can consider as realistic fabrication tolerances 0.01 for the beam splitter transmissivity and 0.01 rad for the active phase control.

**Figure 2:**
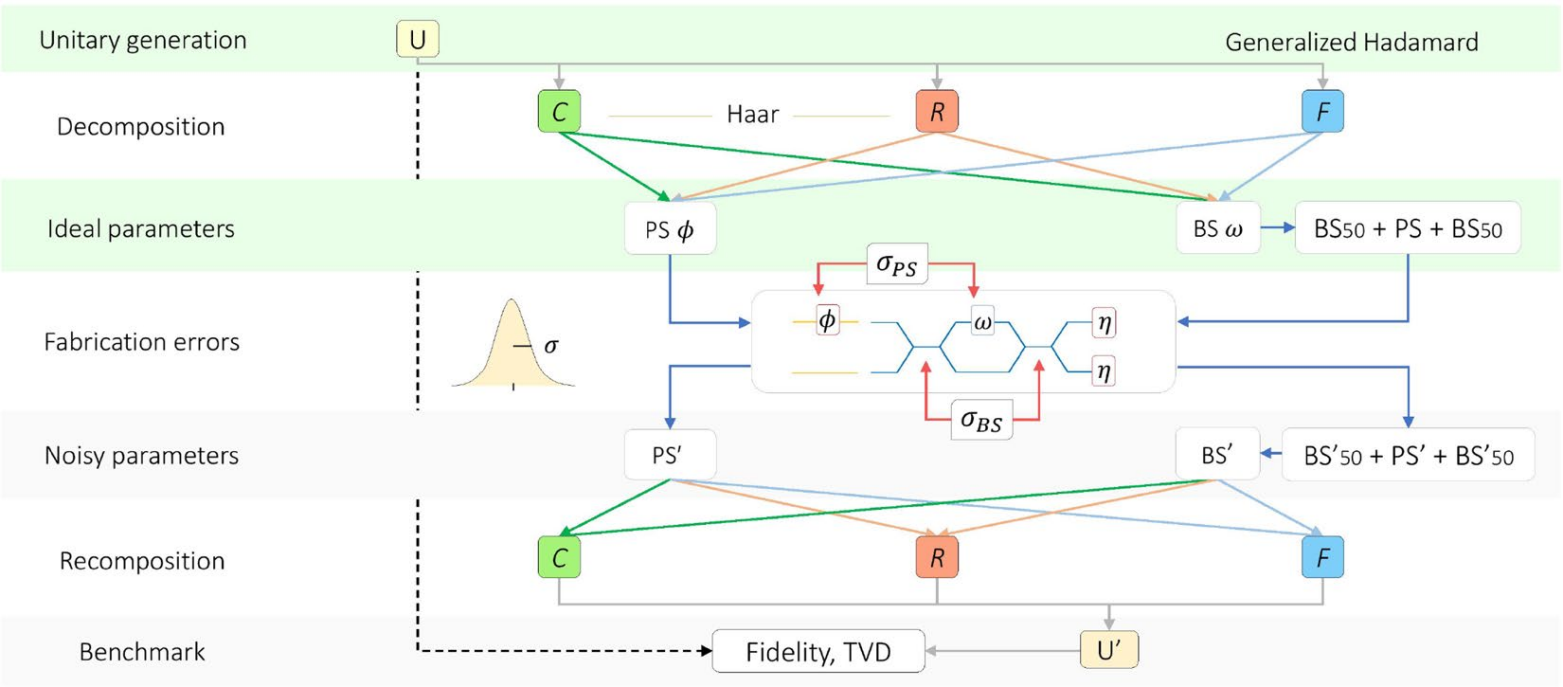
Benchmarking imperfect implementations. Performance of photonic architectures (*C*: Clements^39^, in green; *R*: Reck^38^, in red, *F*: Fast, in blue) is investigated under realistic conditions of noise and losses. Throughout our analysis, simulations are carried out by introducing gaussian noise separately on both phase shifters (*σ*_*PS*_) and beam splitters (*σ*_*BS*_) in their Mach-Zehnder implementation. As figure of merit for their performance we adopt the fidelity^39^ and the total variation distance (TVD) to address more general applications involving also multiphoton evolutions.

Following the scheme of Fig. 2, our investigation on the performances of the three architectures was carried out by introducing various levels of noise on the optical elements and losses after each unit cell. Our Monte Carlo simulations proceed through the following steps:

1. Sample a unitary transformation *U* according to the Haar measure;
2. Apply *C* or *R* algorithms to retrieve the parameters (*ω*_*i*_, *ϕ*_*i*_) according to Eq. (1);
3. Implement each ${T}_{k,k+1}^{(i)}$ as a Mach-Zehnder with input phase shift *ϕ*_*i*_ and *τ*_*i*,1_ = *τ*_*i*,2_ = 2^−1/2^:
  6 $${T}^{(i)}\to -\iota \,(\begin{array}{cc}{\tau }_{i\mathrm{,2}} & \iota \sqrt{1\,-\,{\tau }_{i\mathrm{,2}}^{2}}\\ \iota \sqrt{1\,-\,{\tau }_{i\mathrm{,2}}^{2}} & {\tau }_{i\mathrm{,2}}\end{array})\,(\begin{array}{cc}{e}^{-\iota {\omega }_{i}} & 0\\ 0 & {e}^{\iota {\omega }_{i}}\end{array})\,(\begin{array}{cc}{\tau }_{i\mathrm{,1}} & \iota \sqrt{1\,-\,{\tau }_{i\mathrm{,1}}^{2}}\\ \iota \sqrt{1\,-\,{\tau }_{i\mathrm{,1}}^{2}} & {\tau }_{i\mathrm{,1}}\end{array})\,(\begin{array}{cc}1 & 0\\ 0 & {e}^{\iota {\varphi }_{i}}\end{array})$$
4. Introduce gaussian noise on the four parameters (*τ*_*i*,1_, *τ*_*i*,2_, *ω*_*i*_, *ϕ*_*i*_), by sampling new values from a normal distribution centered on the ideal ones and with widths *σ*_*BS*_ and *σ*_*PS*_ respectively for (*τ*_1_, *τ*_2_) and for (*ω*, *ϕ*);
5. Generate new unit cells from the noisy values, adding possible losses diag(*η*_*i*_) at the output, and rebuild the noisy *U*.

This simple procedure allows us to investigate the effect of noise and losses on the *C*- and *R*-designs for the implementation of Haar random unitaries. Incidentally, our numerical simulations confirm^60^ that predictions would be remarkably different if, instead of generating every time a new unitary according to the Haar measure, we directly generated sets of uniformly distributed random parameters (*ω*_*i*_, *ϕ*_*i*_) to -mistakenly- speed up the calculation. Subsequently, the same analysis is repeated for all three architectures (*C*, *R* and *F*) focusing on the implementation of the Fourier and the Sylvester transformation.

### Benchmarking Haar-random interferometers

**Figure 3:**
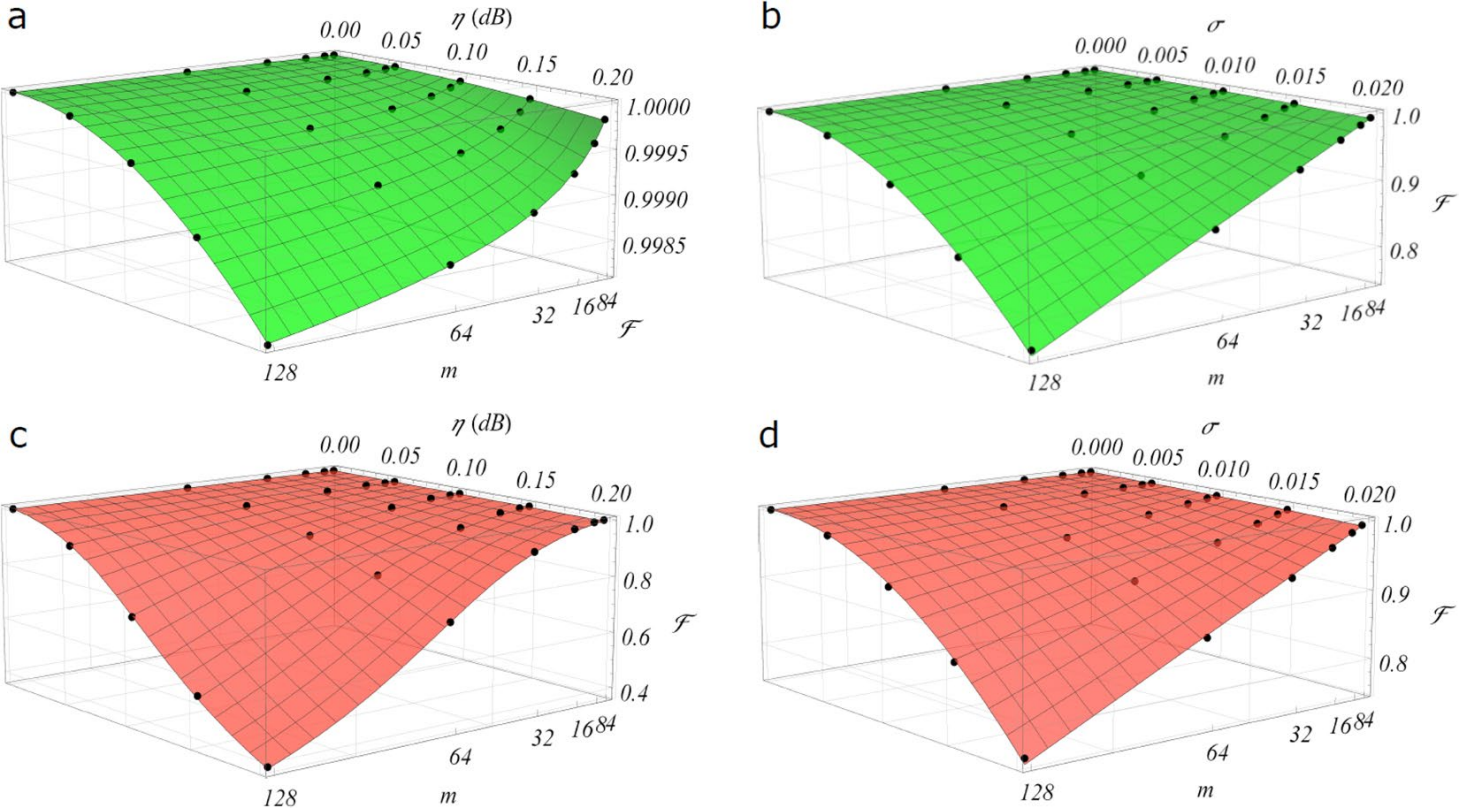
Haar-random with losses and noise. Noise and losses affect the implementation of Haar-random unitaries in different ways in the *C*- and *R*-designs. (**a**,**c**) Average fidelity *F* for different levels of loss *η* per beam splitter and size *m* of the interferometer in the *C* (**a**) and *R* (**c**) designs. Note the difference in vertical scale, due to the more balanced structure of *C* where the dependency on *η* arises from the slight path asymmetry of the outer waveguides. (**b**,**d**) Average fidelity *F* for different levels of noise *σ* in the optical elements and size *m*, averaged over 500 noisy unitaries. Here noise is treated equally on both beam splitters and phase shifters, namely *σ* = *σ*_*BS*_ = *σ*_*PS*_. Note that the scaling is identical in (**b**,**d**) since, averaging over the unitaries, the deterioration of *F* is due to the number of noisy elements, which is the same in the two schemes. Surfaces: heuristic non-linear fits of the data (see Supplementary Note 1).

The results of our first analyses are shown in Fig. 3. The figure of merit adopted to compare the two architectures is the fidelity^39^ ${\mathcal F} ={|\tfrac{{\rm{Tr}}({U}_{imp}^{\dagger }U)}{\sqrt{m{\rm{Tr}}({U}_{imp}^{\dagger }{U}_{imp})}}|}^{2}$, being *U* a *m* × *m* unitary transformation and *U*_*imp*_ its imperfect implementation. Thanks to the rescaling factor in the denominator, accounting for the contribution of lossy implementations, this fidelity is particularly suitable to characterize non-unitary transformations. Figures 3a,b show the deterioration of ${\mathcal F}$ for increasing values of losses *η* per unit cell and size *m* of the circuit for the *C*- and *R*- designs. In this simulation, optical elements are assumed to be immune to fabrication noises in order to isolate the *η*-contribution. As pointed out in ref.^39^, the *C*-design is more resilient to internal losses than the *R*- design, thanks to the almost total balance between the mode interactions. Moreover, as the heuristic non-linear fit suggests (see Supplementary Note 1), also the scaling of the fidelity is much more favorable for the former architecture, features that pushes *C* as a promising candidate for large-scale platforms or where, due to technical issues inherent to the specific implementation, it is not possible to guarantee a low level of losses in each optical element. We observe that, as already pointed out in refs^26,39^, inserting additional lossy elements in the *R*-scheme allows to compensate for the imbalance, at the price of significantly increasing the total amount of losses (see Supplementary Note 4). Figures 3c,d show instead the average effect of a noisy implementation of the optical components over the fidelity. Similarly to the previous analysis, our simulation is carried out for various levels of noise *σ* and different sizes *m*, aiming to retrieve a more complete feeling of its scaling. Noise is assumed to be of equal intensity at this stage on both beam splitters transmissivities (*σ*_*BS*_) and phase shifts (*σ*_*PS*_), i.e. *σ* = *σ*_*BS*_ = *σ*_*PS*_, in order to capture the scaling of the performance in a unique three-dimensional plot. Our simulations confirm previous qualitative estimates^39^ concerning the similarity of the scalings of the average fidelity in the two architectures, providing a clear and quantitative picture of its dependency on the fabrication noise over the optical elements. Such a similar trend is observed also when investigating their performance in the case of crosstalk between thermal shifters (see Supplementary Note 5).

**Figure 4:**
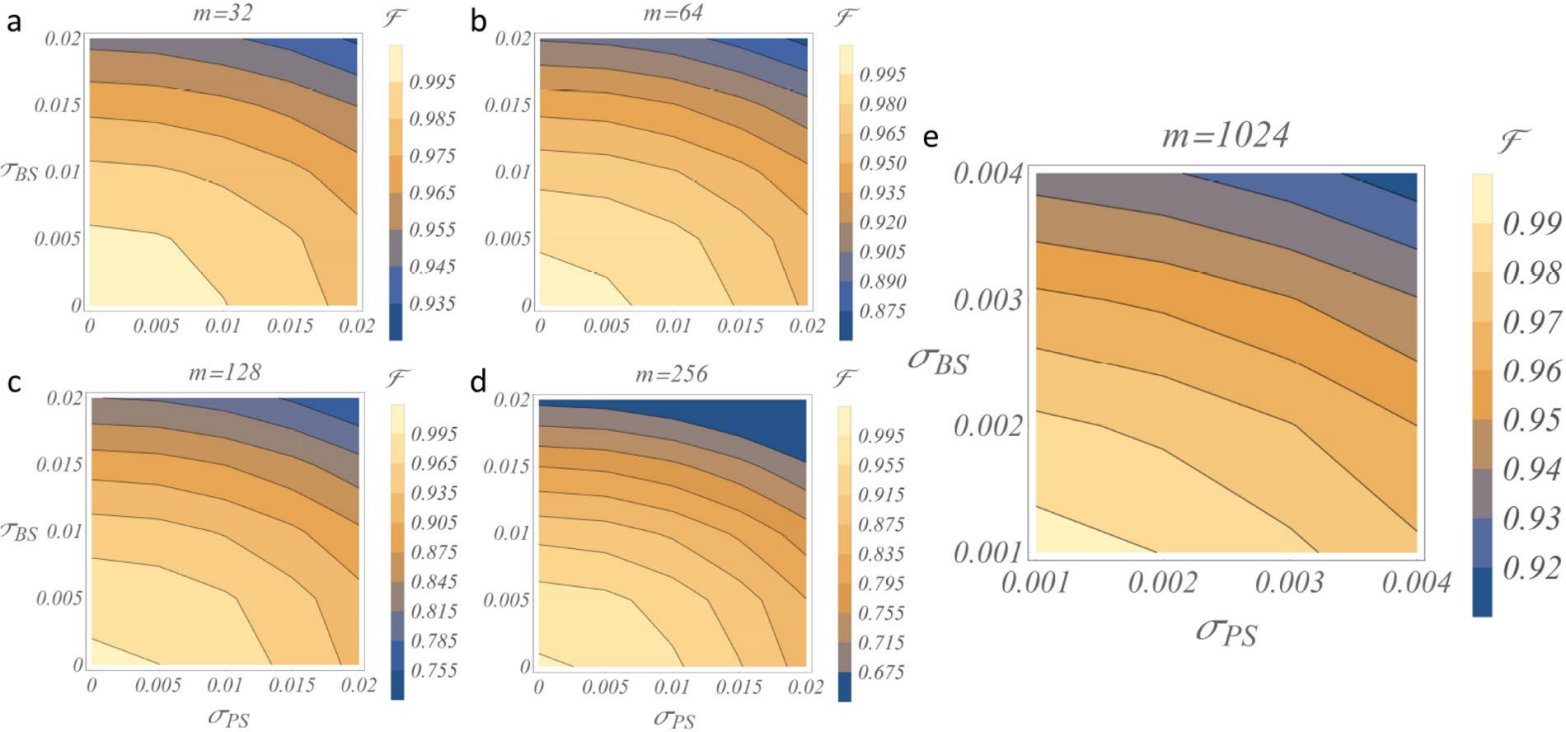
Haar-random with non-ideal transmissivities and phase shifts. Fabrication imperfections and errors in real-time control over the phases are in general on different scales, depending for instance if we are considering (un)balanced beam splitters or (not-)reconfigurable phase shifters. Here noisy Haar-random implementations are investigated under different values of *σ*_*BS*_ and *σ*_*PS*_, to analyze the separate contribution of each source of error to the final unitary transformation. Since the average robustness against noise of the *C*- and *R*-designs is equivalent (see Fig. 3), here the contour plots are shown for the *C* scheme for *m* = 32 (**a**), 64 (**b**), 128 (**c**), 256 (**d**) and 1024 (**e**) (see Supplementary Note 2 for the optimized decomposition adopted for these simulations).

After this preliminary stage, more general investigations have been carried out by considering different levels of noise over the fabrication transmissivities and phases, i.e. studying the practical situation where *σ*_*BS*_ ≠ *σ*_*PS*_. Results of this analysis are shown in the contour plots of Fig. 4 for different sizes of the interferometers, highlighting a number of interesting features. First, the qualitative dependency on the noise seems to remain fixed while increasing the dimension of the circuit, though the average fidelity rapidly drops to low values already at *m* = 64, for intensities of noise that are within the tolerances of current technology, in particular for reconfigurable circuits. Moreover, we observe that the two sources of noise affect the fidelity in a similar way, with an intensity approximately double for the one on the transmissivities in the Mach-Zehnder. Thus, while dynamic control over the phases can in principle mitigate the effects of noisy implementations, non-ideal values of the transmissivities of the symmetric beam splitters remain critical when looking at large-scale implementations. To focus our analysis on the circuit size allowed by current technology, we have considered as a reference current top-level implementations for quantum applications. Circuits with up to *m* = 16 have been reported for non-reconfigurable femtosecond laser written circuits^59^, *m* = 6 for universally reconfigurable lithographic circuits^24^ and *m* = 26 for reconfigurable lithographic circuits that do not implement a fully arbitrary unitary matrix^25^. Reasonable sizes achievable in the near future thus can be *m* = 32 or *m* = 64 depending on the level of reconfigurability required. However, looking at future large-scale implementations, we see that for *m* = 1024 even a very low level of noise makes the average fidelity drop to values as low as ~0.90. Note the shift in the axes scales between the four left contour plots (*m* ≤ 256) and the one on the right (*m* = 1024). Thus, for an efficient scaling a technology leap will be required not only in the circuit size but also on the level of fabrication tolerances.

**Figure 5:**
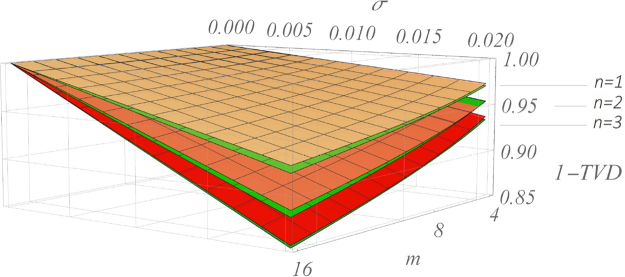
Multiphoton interference in noisy Haar-random interferometers. Several applications require to evolve many-photon states in large-scale quantum circuits. The total variation distance (TVD) is in this sense a good estimate of the goodness of an experimental implementation, being it a natural measure of the distance between two probability distributions^53–56^. Here, plots compare the ideal and noisy output probability distributions relative to *n* = 1, 2, 3-photon collision-free states, averaged over all the inputs. For each *n*, 100 unitaries are sampled and implemented according to the *C*- (green) and *R*- (red) designs setting *σ*_*BS*_ = *σ*_*PS*_ = *σ*. Our analysis confirms on one hand the prediction of Fig. 3c,d, where the *R*-design is found to be slightly more robust againt noise, but on the other it shows how this difference seems to become negligible for increasing values of *n*. Surfaces: heuristic non-linear fits of the data (see Supplementary Note 1).

So far, we adopted as figure of merit the fidelity of the quantum process. Though perfectly suitable to benchmark noisy transformations, this quantity fails in general to highlight more complex many-particle phenomena. This requirement becomes even more strict for instance in the context of Boson Sampling and, more specifically, in its validation, where multiphoton interference plays a key role to guarantee the computational complexity of the problem. Indeed, while Boson Sampling preserves its complexity even in lossy and imperfect conditions below a certain threshold^51,54^, multiphoton interference in Hadamard interferometers^44^ was shown to be a promising tool to correctly validate its operation. Partial deviation from their ideal symmetric structures can then spoil the interference effects in the output distributions. For this reason, we investigated the performance of noisy architectures also in the scope of multiphoton output probability distributions, employing as figure of merit the total variation distance (TVD) between the ideal (*P*) and actual ($\tilde{P}$) *n*-photon distributions: $TVD(P,\,\tilde{P})=\tfrac{1}{2}\,{\sum }_{i}\,|{p}_{i}\,-\,{\tilde{p}}_{i}|$. Indeed, the TVD plays a relevant role in testing statistical hypotheses since the quantity 1 − *TVD* is a lower bound on the sum of Type I and Type II error rates^61^. From a practical point of view, $TVD(P,\tilde{P})$ is also a simple and natural metric to quantify the discrepancy between the two probability distributions *P* and $\tilde{P}$. Results for this analysis are shown in Fig. 5: again, noise affects almost equally the *C* and *R* architectures when averaging over all the input/output states. The slight difference in favor of the *R*-design may not be practically appreciable in real experimental conditions and it rapidly becomes negligible for higher values of (*n*, *m*). Thus, the two architectures behave equally as far as lossless multiphoton investigations are concerned.

We conclude from our analysis that the *C*- and *R*-designs are ultimately equivalent in terms of resilience to noise when averaging over all input/output configurations. On one side, the single optical elements affect in a different way the elements of the unitary transformation in the two schemes, being the *C* and *R* parameters more localized respectively in the upper corner and central part of the unitary matrix. However, for practical noise levels and general multi-input applications, this difference does not give rise to any effective deviation between the two schemes. In contrast, the two architectures behave differently when it comes to lossy implementations, where the symmetric *C*-design outperforms the *R* scheme.

### Benchmarking Fourier and Sylvester interferometers

Though the *C*- and *R*-designs are universal for unitary evolutions, often it is desirable to have circuits optimized for specific relevant tasks. It is the case of the quantum Fourier or Sylvester transforms, which have importance on their own in different scopes of quantum information processing.

**Figure 6:**
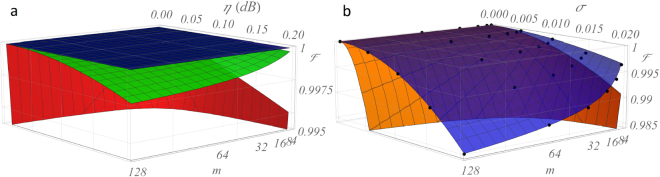
Generalized Hadamard transformations with *C*-, *R*- and *F* designs. While universal for unitary decompositions, the *C*- and *R*- architectures offer suboptimal solutions for the implementation of specific classes with higher symmetries. The Fast architecture is optimized for the implementation of the quantum Fourier transform and the class of Hadamard transforms. (**a**) Average fidelity as a function of losses per unit cell and network size (blue, green and red surfaces for *F*-, *C*- and *R*-designs respectively) considering the Mach-Zehnder implementation for all three architectures. Full symmetry between the optical paths cancels out the effect of constant losses per unit cell in the *F*- design. (**b**) Fast architectures (blue surface) are also more resilient to fabrication noise, thanks to the reduced depth of the circuit. Here, only one (orange) surface is shown for both *C*- and *R*-designs, assuming equal resilience to noise. Surfaces: heuristic non-linear fits of the data (see Supplementary Note 1).

In this section we investigate the performance of the Fast (*F*) scheme^42–46^ for the implementation of generalized Hadamard transformations^58^ and compare it with the average performance of the two universal designs, following the same procedure outlined in Fig. 2. Figure 6 reproduces the analysis of Fig. 3 including the *F* architecture. Being the circuit completely symmetric, the structure is totally immune to constant propagation losses, beating even the highly resilient *C*-design. The *F*-design is also more resilient to noise, thanks to the reduced depth of the circuit which lowers the number of noisy optical elements from $\frac{m}{2}(m-1)$ to $\tfrac{m}{2}\,\mathrm{log}\,m$. A benchmark summary of this comparison is reported in Table 1 for both the quantum Fourier transform and the Sylvester interferometers.

**Table 1:** Benchmarking Hadamard transformations.

Quantum Fourier transform and Sylvester										
		*m* = 64			*m* = 128			*m* = 256		
*σ*_*PS*_	*σ*_*BS*_ →	0.005	0.01	0.02	0.005	0.01	0.02	0.005	0.01	0.02
0.001	F	0.999 (1)	0.995 (1)	0.981 (4)	0.998 (1)	0.994 (1)	0.976 (4)	0.998 (1)	0.992 (1)	0.972 (3)
	C	0.994 (1)	0.975 (1)	0.904 (5)	0.987 (1)	0.950 (1)	0.815 (4)	0.975 (1)	0.903 (1)	0.664 (3)
	R	0.994 (1)	0.975 (1)	0.904 (5)	0.987 (1)	0.950 (1)	0.817 (4)	0.975 (1)	0.903 (1)	0.669 (3)
0.01	F	0.998 (1)	0.994 (1)	0.980 (4)	0.997 (1)	0.993 (1)	0.975 (4)	0.997 (1)	0.992 (1)	0.971 (4)
	C	0.984 (1)	0.966 (2)	0.895 (5)	0.969 (1)	0.932 (2)	0.800 (4)	0.938 (1)	0.869 (1)	0.639 (3)
	R	0.984 (1)	0.966 (2)	0.895 (5)	0.969 (1)	0.933 (2)	0.802 (4)	0.938 (1)	0.870 (1)	0.645 (3)
0.02	F	0.995 (1)	0.991 (1)	0.976 (5)	0.994 (1)	0.989 (1)	0.971 (5)	0.993 (1)	0.988 (1)	0.968 (4)
	C	0.957 (2)	0.939 (3)	0.870 (5)	0.915 (2)	0.881 (3)	0.756 (4)	0.836 (2)	0.775 (2)	0.569 (4)
	R	0.957 (2)	0.939 (3)	0.870 (5)	0.915 (2)	0.881 (3)	0.758 (5)	0.837 (2)	0.777 (3)	0.577 (3)

Generalized Hadamard transformations benefit from optimized architectures, obtained from the efficient algorithms^40,42^ developed for the fast quantum Fourier transform. Here, average Fidelities are reported for each combination of size *m* and noises *σ*_*BS*_, *σ*_*PS*_, together with the uncertainty on the last digits, as estimated with a Monte Carlo simulation over 500 noisy unitaries. Data for the Fourier and Sylvester transformations are displayed in a single table since the values in the two cases are equal within a discrepancy lower than ~0.001.

## Conclusions

Photonic technologies promise to enable the application of several quantum information protocols, ranging from fundamental research to quantum computation and optical quantum networks. In this work we have provided a comprehensive analysis of the performance of the three main interferometric schemes, namely the triangular^38^ and square^39^ designs and the Fast architecture, under realistic conditions of losses and noise. Our investigations quantitatively address the issue of imperfect implementations for interferometers of increasing dimension, aiming to embrace both mid-term and long-term technological standards. Our results confirm the qualitative expectation that the square design performs way better, in terms of fidelity, between ideal and lossy evolutions with respect to the triangular one, even though the latter exhibits a slightly enhanced resilience to fabrication noise when considering also the total variation distance. Thus, we conclude that the square design is preferable in practical applications involving multi-input protocols and Haar-random generation, especially in high-dimension circuits where the issue of propagation losses becomes critical.

Fast architectures represent instead a specialized design to implement a significant class of unitary evolutions, highly optimized for the realization of Fourier and Sylvester quantum transformations. Our results quantitatively highlight the improved performance of this scheme with respect to the universal ones, thus making it the preferred choice when applicable. Furthermore, we have provided a closed-form expression for the elements of the 2^*n*^-dimensional unitary describing these circuits, which allows to suitably tailor single elements of the unitary transformations mapping them to the transmissivities and the phase shifts in the real optical implementation. Due to the deep relevance of this broad class of quantum routines, and thanks to the high enhancements offered by their optimized implementations, we expect Fast architectures to gain a key role among photonic platforms in synergy with the universal schemes, to fully benefit from the unique advantages of both designs.

During the completion of this manuscript, related work has been reported in refs^60,62^.

### Data availability

The datasets generated and analysed during the current study are available from the corresponding author on reasonable request.

## Electronic supplementary material

Supplementary Information
