# Supplementary material for: Benchmarking integrated linear-optical architectures for quantum information processing
Source: Sci Rep. 2017 Nov 9;7:15133. doi: 10.1038/s41598-017-15174-2 (PMC5680265; doi:10.1038/s41598-017-15174-2)
Supplement: Supplementary file 1 — Supplementary Information [file 41598_2017_15174_MOESM1_ESM.pdf]

Supplementary Information
Benchmarking integrated linear-optical architectures for quantum information processing

Fulvio Flamini,[1,*] Nicolò Spagnolo,[1] Niko Viggianiello,[1] Andrea Crespi,[2,3] Roberto Osellame,[2,3] and Fabio Sciarrino[1]

[1]*Dipartimento di Fisica, Sapienza Università di Roma, Piazzale Aldo Moro 5, I-00185 Roma, Italy*
[2]*Istituto di Fotonica e Nanotecnologie, Consiglio Nazionale delle Ricerche (IFN-CNR), Piazza Leonardo da Vinci, 32, I-20133 Milano, Italy*
[3]*Dipartimento di Fisica, Politecnico di Milano, Piazza Leonardo da Vinci, 32, I-20133 Milano, Italy*

SUPPLEMENTARY NOTE 1: DETAILS ON THE NON-LINEAR FITS

Below we provide details on the non-linear heuristic fits shown in the main text. Functions are chosen by selecting the dependency that best matched the simulated data points in the region under investigation, while keeping low the number of free parameters to avoid overfitting and minimizing their p-values. Though various functions yield behaviors compatible with the data in the region investigated, the p-value check always highlighted undesired symptoms until the best dependency is found. All functions reported passed this test and most of them have been guessed with knowledge about the physical quantity. The Fidelity $\mathscr{F}$ as a function of the size of the circuit m and loss per beam splitter η (in dB) has the following expressions, respectively for the C- [S1] and R-designs [S2]

$$\mathscr{F}_C(m,\eta) \sim 1 - A_c\,\eta^2\,\log(B_c\,m + C_c) \qquad A_c = 0.0158 \pm 0.0002, \quad B_c = 0.140 \pm 0.005, \quad C_c = 0.79 \pm 0.01 \tag{S1}$$

$$\mathscr{F}_R(m,\eta) \sim \frac{A_r + e^{B_r\,m\,\eta}}{A_r + e^{C_r\,m\,\eta}} \qquad A_r = 7.55 \pm 0.51, \quad B_r = 0.163 \pm 0.007, \quad C_r = 0.204 \pm 0.007 \tag{S2}$$

as retrieved from a non-linear fit in the region $m \in [4, 256]$ and $\eta \in [0, 0.2]$, by sampling 500 Haar-random lossy unitaries for each simulated point. Similarly, $\mathscr{F}$ as a function of m and noise σ has the expressions (F: Fast scheme)

$$\mathscr{F}_C(m,\sigma) \sim 1 - \alpha\,m\,\sigma^2 \qquad \alpha = 4.932 \pm 0.034 \tag{S3}$$

$$\mathscr{F}_R(m,\sigma) \sim 1 - \beta\,m\,\sigma^2 \qquad \beta = 4.896 \pm 0.036 \tag{S4}$$

$$\mathscr{F}_F(m = 2^n,\sigma) \sim 1 - \gamma\,n\,\sigma^2 \qquad \gamma = 7.73 \pm 0.04 \tag{S5}$$

as retrieved from a non-linear fit in the region $m \in [4, 128]$ and $\sigma \in [0, 0.02]$, by sampling 500 Haar-random noisy unitaries for each simulated point. We observe that the effective dependency of $\mathscr{F}$ is indeed on the depth of the circuit, i.e. $O(m)$ for C and R, $O(\log m)$ for F. Finally, the 3-photon total variation distance as a function of m and σ has the expression

$$TVD^{(3)}_{C,R}(m,\sigma) \sim 1 - A_c\,\sigma\,\sqrt{m(m + B_c)} \qquad A_c = 0.23 \pm 0.01, \quad B_c = 47 \pm 5 \tag{S6}$$

which is found to be approximately the same for both C and R schemes. The formula is estimated in the region $m \in [4, 16]$ and $\sigma \in [0, 0.02]$, by sampling 100 Haar-random noisy unitaries for each simulated point.

SUPPLEMENTARY NOTE 2: SQUARE DECOMPOSITION WITH HIGH-DIMENSIONAL UNITARIES

Below we provide a sketch of the routine adopted for the C decomposition of high-dimensional unitaries [S1]. Our routine is formally equivalent to the original algorithm but it avoids matrix multiplications, thus significantly decreasing the computational resources required for large interferometers. Steps marked with (*) can be implemented easily without a full matrix multiplication, since each T matrix affects only one pair of rows/columns of U.

* fulvio.flamini@gmail.com

First, following [S1], we retrieve the $\frac{m(m-1)}{2}$ pairs of parameters $\{(\phi,\omega)\} \cup \{(\phi^\dagger,\omega^\dagger)\}$

For i = 1 ... m

if i == odd: For j = 0 ... i - 1

$$\phi_s^\dagger = \arg(U_{m-j,i-j}) - \arg(U_{m-j,i-j+1})$$
$$\omega_s^\dagger = \text{arctg}\left(\frac{U_{m-j,i-j+1}}{U_{m-j,i-j}}\, e^{\imath\phi_s^\dagger}\right)$$
$$U \to U\,.\,T^\dagger_{i-j,i-j+1}\left(\phi_s^\dagger,\omega_s^\dagger\right) \qquad (*)$$

else: For j = 0 ... i - 1

$$\phi_s = \arg(U_{m+j-i+1,j+1}) - \arg(U_{m+j-i,j+1})$$
$$\omega_s = \text{arctg}\left(\frac{U_{m+j-i,j+1}}{U_{m+j-i+1,j+1}}\, e^{\imath\phi_s}\right)$$
$$U \to T_{m+j-i,m+j-i+1}\left(\phi_s,\omega_s\right).\,U \qquad (*)$$

Then we need to move the remaining diagonal matrix D to the left of the decomposition [S1], which leads us to a new set of parameters $\{(\phi,\omega)\}$. By defining $\delta = \{\arg(\text{diag}\, D)\}$ and χ as the $\left(\frac{m(m-1)}{2}\times 2\right)$-dimensional list of pairs of modes mixed step by step by the $N = \sum_{i=0}^{\lceil\frac{m}{2}\rceil-1}(2i)$ matrices T (as given by the order of the decomposition), we iteratively update the $\{\phi\}$ as

For $s = 1 \ldots N$

$$tmp = \delta_{\chi_{s,2}}$$
$$\delta_{\chi_{s,2}} = \delta_{\chi_{s,1}} + \phi_s$$
$$\phi_s = \delta_{\chi_{s,1}} - tmp$$

The final list of parameters π is then $\quad \pi = \{(\phi,\omega)\} \cup \text{Reversed}\{(\phi^\dagger,\omega^\dagger)\}$,
where *Reversed* takes the list of pairs of parameters $\{(\phi_s^\dagger,\omega_s^\dagger)\}_{s=1\ldots n}$ in the reversed order: $\{(\phi_s^\dagger,\omega_s^\dagger)\}_{s=n\ldots 1}$.

SUPPLEMENTARY NOTE 3: TOTAL VARIATION DISTANCE AS FIGURE OF MERIT

The Fidelity of a quantum circuit with its ideal operation is a natural choice for the figure of merit of an architecture, since it usually does not depend on factors external to the structure to characterize. However, the Fidelity does not give complete insight on relevant trends in the performance of a realistic circuit for multiphoton applications. In this sense, though depending also on additional factors such as purity and degree of distinguishability between the input photons, the Total Variation Distance is a useful indicator of the goodness of a multiphoton operation, as a measure of the closeness of the n-photon output probability distribution with the one desired. Supplementary Fig.S1 reports on a simple simulation to clarify this aspect. In our analysis we simulated m=8-dimensional noisy circuits injected with n=2,3,4,5 photons and calculated, for each of 1000 Haar-random unitaries, their Fidelities and TVDs. As we can see in the 3D smooth histograms, the Fidelity remains bounded in a narrow region (depending on the amount of noise introduced in the circuit, here $\sigma = 0.02$) while the TVD between the ideal and noisy implementations increases with n. Though hard to probe with numerical simulations, this effect might be even amplified for larger m and n.

SUPPLEMENTARY NOTE 4: COMPENSATING FOR UNBALANCED LOSSES IN THE RECK-SCHEME

Though natively unbalanced, the average Fidelity with the R-design can be enhanced to the level of the C-design by inserting additional lossy elements on the straight waveguides. We have carried out simulations to investigate on this possibility, inserting artificial losses of different intensity along the input/output modes and estimating the average Fidelity for each trial. While the optimal loss intensity is in-principle unknown, numerical simulations confirm the expectation that they simply need to increase linearly along the optical modes, due to (and exactly compensating for) the reduced number of lossy elements on that line. Supplementary Fig.S2 shows the average Fidelity for the R-design (red), C-design (green) and R with nearly-optimal linear

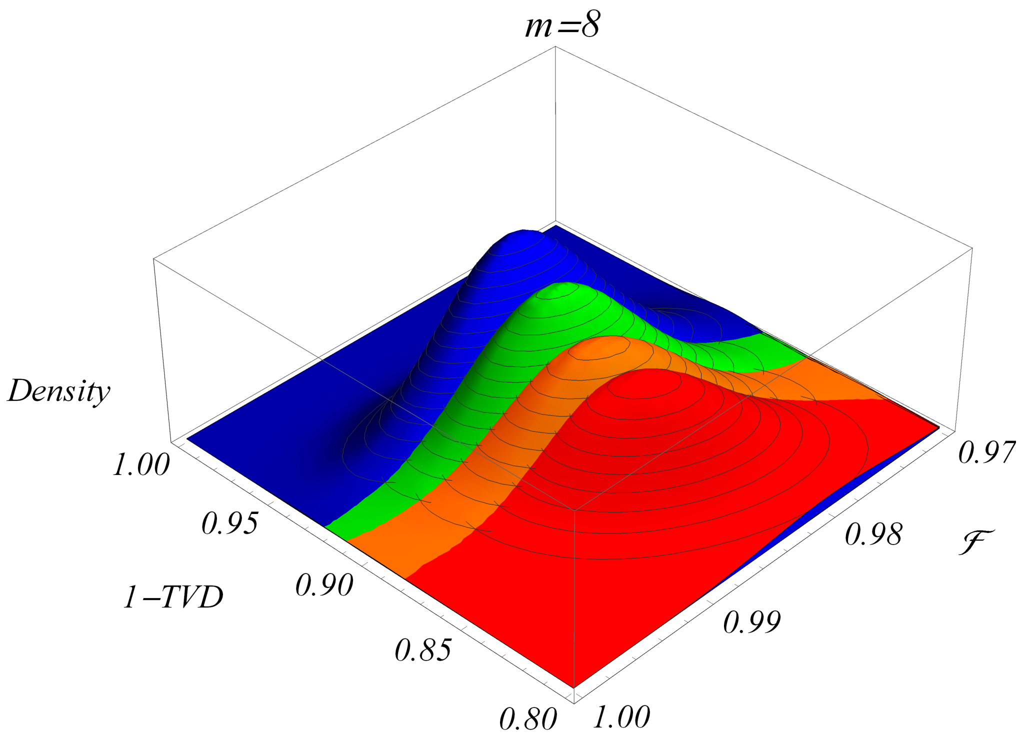

Supplementary Figure S1. **Total Variation Distance against Fidelity**. 3D smooth Gaussian histograms representing the Fidelity $\mathscr{F}$ versus the Total Variation Distance (TVD) as retrieved from 1000 noisy 8-dimensional Haar-random circuits. We see that the Fidelity remains bounded in a narrow region while the TVD increases for $n = 2$ (blue), $n = 3$ (green), $n = 4$ (orange) and $n = 5$ (red) photons.

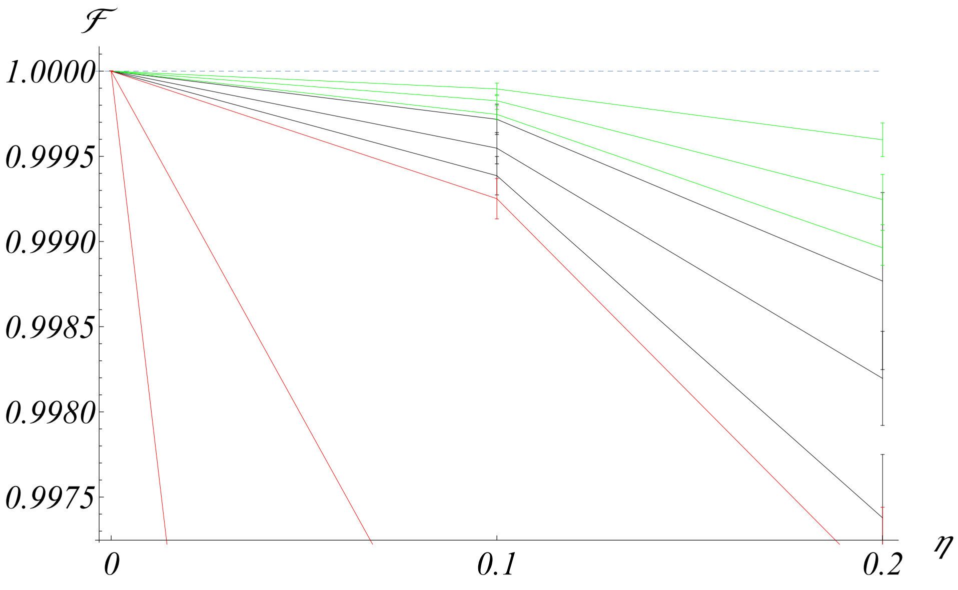

Supplementary Figure S2. **Enhancing Fidelity in the R-scheme**. Fidelities averaged over 100 Haar-random unitaries with the R scheme (red), C scheme (green) and R with artificial losses (black) for $m = 8, 16, 32$ modes. For all schemes: the higher m, the lower $\mathscr{F}$.

artificial losses (black). From our analysis we conclude that the R-scheme can in principle account for its inherent imbalance in the loss distribution, at the cost of adding a total loss for an amount equal to that already present due to the non-ideal fabrication. Thus, while compensating for unbalanced loss distribution, such approach with the R-scheme leads to a larger amount of overall losses then the C one with the same number of modes.

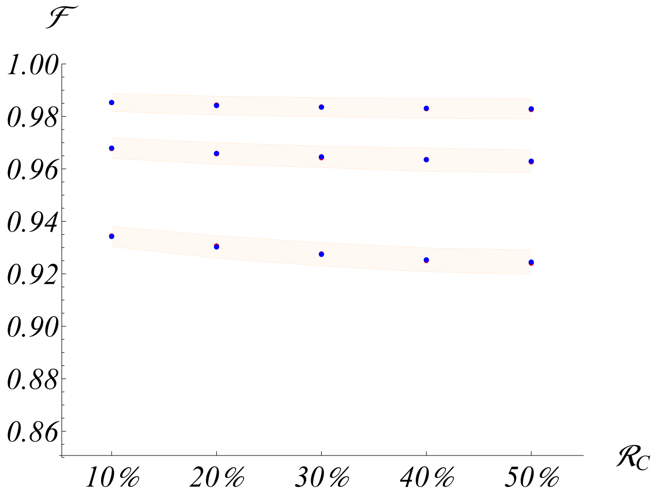

(a)

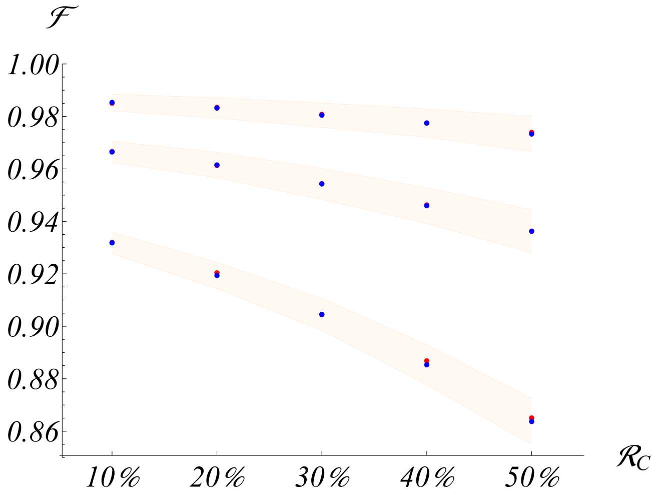

(b)

Supplementary Figure S3. **Crosstalk in R- and C- schemes**. Effects of crosstalk on circuits of size $m = 8, 16, 32$ with the Reck scheme (red points) and Clements scheme (blue points), averaged over 500 fraction R_c of elements with crosstalk, where the noise on each element does not depend (a) or depends proportionally (b) on the number of neighbors with crosstalk. Orange regions: 1-sigma band (almost equivalent for the two schemes) around the mean values. For all schemes: the higher m, the lower $\mathscr{F}$.

SUPPLEMENTARY NOTE 5: EFFECTS OF CROSSTALK

Possible effects of crosstalk between thermal shifters are a relevant issue in practical implementations.To quantify this aspect we have carried out numerical simulations using two simple models:

1. With the first approach (Supplementary Fig.S3a), we start from the decomposition of a Haar-random unitary and randomly pick up a different fraction of thermal shifters (in both beam splitters and phase shifters), that are progressively supposed to affect the behavior of its neighbors. For all the optical elements in the neighborhood of a picked up thermal shifter, we then introduce a Gaussian noise about the ideal value of the phase and measure the Fidelity with the ideal unitary.

2. With the second approach (Supplementary Fig.S3b), we again pick up a random set of elements but apply a Gaussian noise proportional to the number of shifters introducing a crosstalk.

For both simulations, we plot the average Fidelity for the R- (red) and C- (blue) designs retrieved from 1000 Haar-random unitaries, for varying fractions R_c of elements with crosstalk. The two curves present a similar scaling, as discussed in the main text for noisy implementations. Moreover, the R scheme is again found to be (on average) slightly more resilient to noise than the C one, though this difference is negligible for practical applications. Furthermore, we expect this difference to vanish for larger dimensions, since this effect depends mainly on boundary elements whose fraction linearly goes to zero. In the plots, error bars (almost equal for the two schemes) and orange regions represent 1-sigma standard deviations for the 1000 samples.

SUPPLEMENTARY REFERENCES

[S1] W. R. Clements, P. C. Humphreys, B. J. Metcalf, W. S. Kolthammer, and I. A. Walmsley, Optica **3**, 1460-1465 (2016).
[S2] M. Reck, A. Zeilinger, H. J. Bernstein, and P. Bertani, Phys. Rev. Lett. **73**, 58 (1994).
